# Supplementary material for: Primary metabolism determines the outcome of salicylic acid-mediated immune induction
Source: bioRxiv. 2025 Oct 14:2025.10.13.682132. Preprint. [Version 1] doi: 10.1101/2025.10.13.682132 (PMC12632896; doi:10.1101/2025.10.13.682132)
Supplement: 1 [file NIHPP2025.10.13.682132v1-supplement-1.pdf]

bioRxiv preprint doi: https://doi.org/10.1101/2025.10.13.682132; this version posted October 14, 2025. The copyright holder for this preprint (which was not certified by peer review) is the author/funder, who has granted bioRxiv a license to display the preprint in perpetuity. It is made available under aCC-BY 4.0 International license.

SUPPLEMENTAL INFORMATION

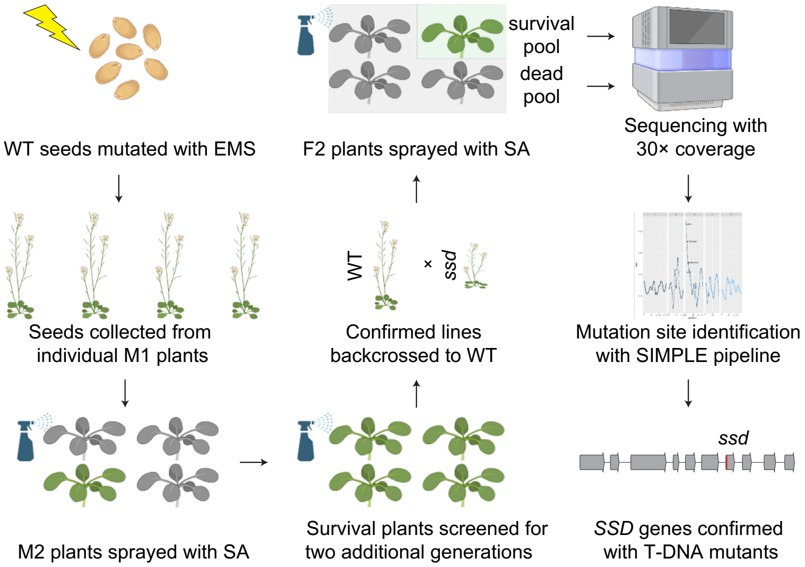

Extended Data Fig. 1. Workflow for the genetic screen for *ssd* mutants.

WT seeds were mutagenized with EMS and sown in soil. Seeds from each M1 plant were collected separately, germinated in soil and grown for 14 days under LD cycles before treatment with 1 mM SA at the end of dark cycle. After 72 hours under DD, the surviving plants from each M2 line were collected individually and retested through two additional selfing generations. Selected surviving plants from each line were then backcrossed with WT plants to generate the F1 progeny. For each putative *ssd* line, approximately 60 surviving and 100 dead F2 plants were collected for bulk segregant whole genome sequencing at 30-fold coverage of the Arabidopsis genome. Sequencing data were analyzed using the SIMPLE pipeline to identify candidate mutations, from which the causal mutation was confirmed by examining the death rate of corresponding T-DNA insertion mutants.

bioRxiv preprint doi: https://doi.org/10.1101/2025.10.13.682132; this version posted October 14, 2025. The copyright holder for this preprint (which was not certified by peer review) is the author/funder, who has granted bioRxiv a license to display the preprint in perpetuity. It is made available under aCC-BY 4.0 International license.

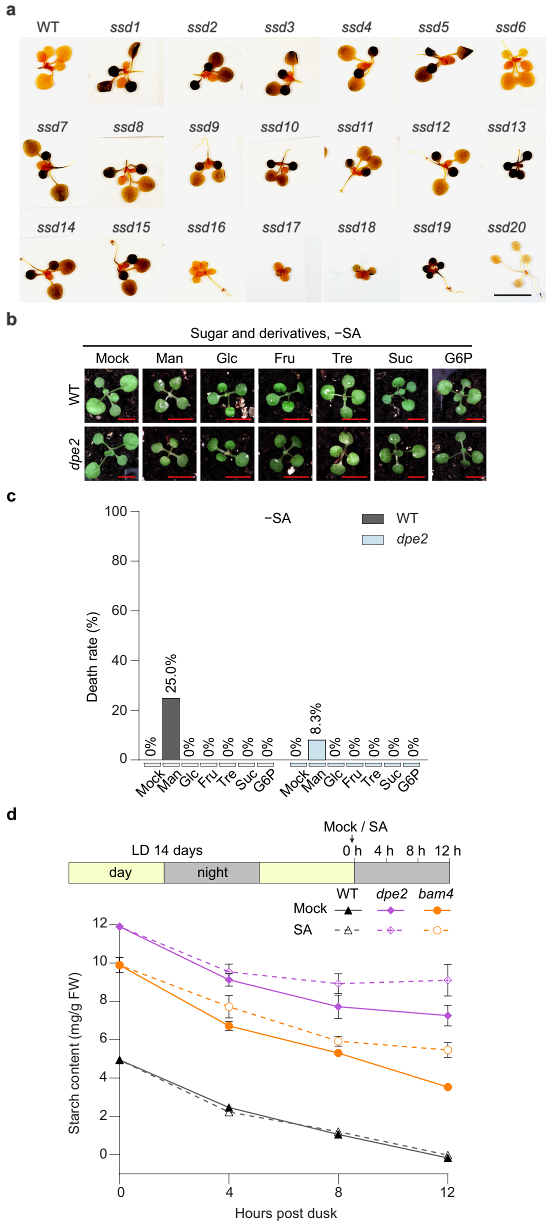

Extended Data Fig. 2. Starch measurement and SA-induced plant death rate assessments under control conditions.

(a) Starch staining in WT and all the *ssd* mutants at dawn. Scale Bar = 1 cm. Plants grown for 14 days under LD cycles were collected at dawn and stained using the Lugol reagent. (b)

bioRxiv preprint doi: https://doi.org/10.1101/2025.10.13.682132; this version posted October 14, 2025. The copyright holder for this preprint (which was not certified by peer review) is the author/funder, who has granted bioRxiv a license to display the preprint in perpetuity. It is made available under aCC-BY 4.0 International license.

Representative images of the plants treated with sugar and their derivatives without SA. Plants grown the same way as the genetic screen were sprayed with mock or 100 mM mannitol (Man), sugars and sugar derivatives following the dark period and kept in DD for 72 h before imaging. Glu, glucose; Fru, fructose; Tre, trehalose; G6P, glucose-6-phosphate; Scale bar = 0.5 cm. (c) Quantification of the death rate for the plants in (b). (d) Effects of SA on the starch degradation rate in WT, *dpe2*, and *bam4* mutants. Plants grown under the LD conditions for 14 days were sprayed with mock or 1 mM SA following the light cycle and samples were collected for starch quantification at 0, 4, 8, 12 h with mock or SA treatment in the dark. All data are presented as mean ± s.e.m. Six biological replicates were performed for each sample.

bioRxiv preprint doi: https://doi.org/10.1101/2025.10.13.682132; this version posted October 14, 2025. The copyright holder for this preprint (which was not certified by peer review) is the author/funder, who has granted bioRxiv a license to display the preprint in perpetuity. It is made available under aCC-BY 4.0 International license.

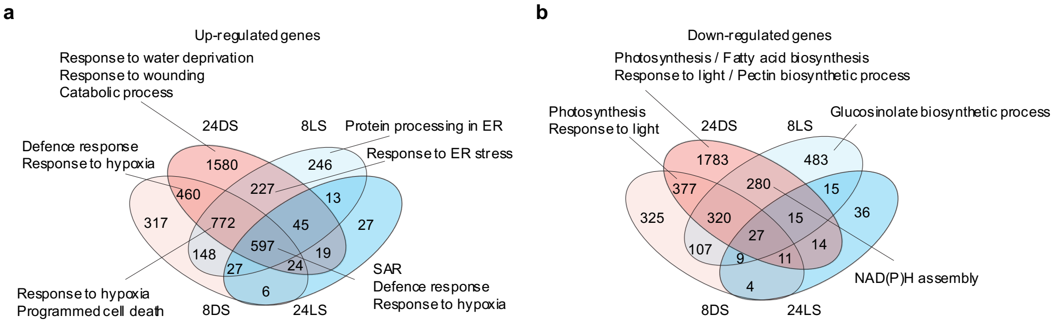

Extended Data Fig. 3. Transcriptome analysis of SA and glucose effects on plants under LD and DD conditions.

(a and b) Venn diagrams showing the overlap of SA upregulated (a) and downregulated (b) DEGs between LD and DD conditions by comparing SA- to mock-treated samples at the corresponding timepoints and light conditions. Samples in this figure are designed by: timepoint (8, 24) h, light condition (D for DD; L for LD), and treatment versus mock (S, SA versus mock; G, glucose versus mock; SG, SA+glucose versus mock). DEGs were defined with Padj < 0.05 and LFC > 1.

bioRxiv preprint doi: https://doi.org/10.1101/2025.10.13.682132; this version posted October 14, 2025. The copyright holder for this preprint (which was not certified by peer review) is the author/funder, who has granted bioRxiv a license to display the preprint in perpetuity. It is made available under aCC-BY 4.0 International license.

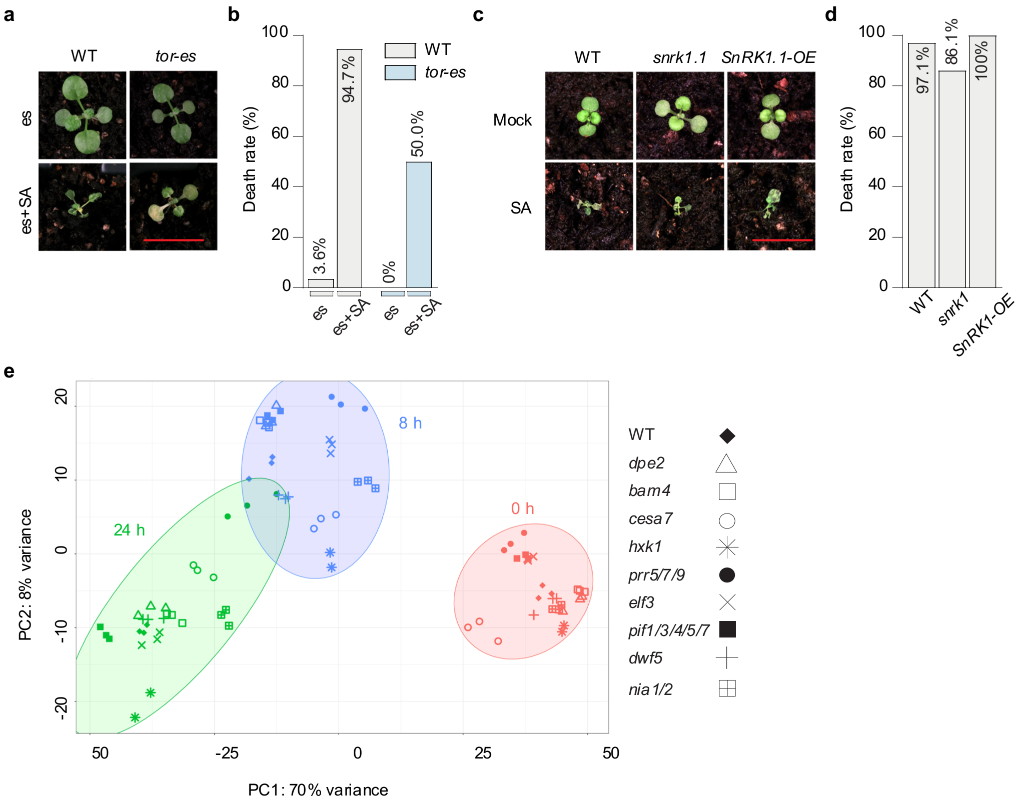

Extended Data Fig. 4. Phenotypes for sugar signalling-related mutants and PCA analysis of transcriptome data from survival mutants.

(a) Representative images of WT and the *tor-es* (the β-estradiol inducible silencing line for *TOR*) plants after mock or SA treatment. Plants grown under LD cycles were pre-sprayed with 10 μM β-estradiol (es) for 5 days (one time each day) until day 14 and then treated with mock or 1 mM SA following the dark cycle. Then the plants were kept in darkness for 72 h before imaging. (b) Quantification of the death rate for the plants in (a). (c) Representative images of WT, the *snrk1.1* mutant and *SnRK1.1-OE* plants with mock or SA treatment. Plants grown for 14 days under LD cycles were sprayed with mock or 1 mM SA following the dark cycle and kept in darkness for 72 h before imaging. (d) Quantification of the death rate for the plants in (c). (e) PCA of WT and survival mutant transcriptomes with SA treatment in DD. Representative gene mutant plants were

bioRxiv preprint doi: https://doi.org/10.1101/2025.10.13.682132; this version posted October 14, 2025. The copyright holder for this preprint (which was not certified by peer review) is the author/funder, who has granted bioRxiv a license to display the preprint in perpetuity. It is made available under aCC-BY 4.0 International license.

grown as in (c) and collected at 0 h, 8 h and 24 h after SA treatment in DD. Samples separated in a temporal manner in the PCA plot are marked by ellipses. Scale bar = 1 cm.

bioRxiv preprint doi: https://doi.org/10.1101/2025.10.13.682132; this version posted October 14, 2025. The copyright holder for this preprint (which was not certified by peer review) is the author/funder, who has granted bioRxiv a license to display the preprint in perpetuity. It is made available under aCC-BY 4.0 International license.

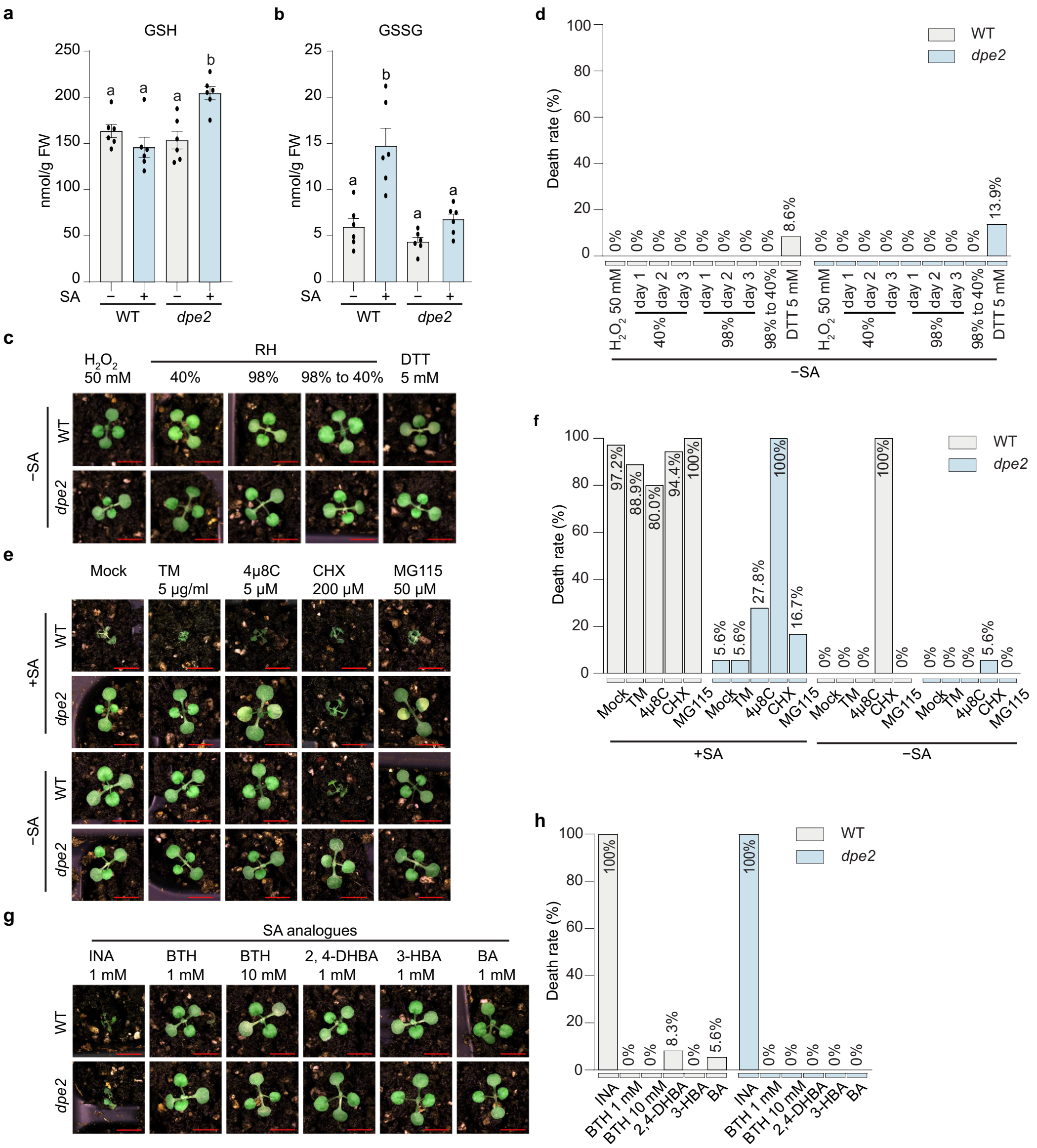

Extended Data Fig. 5. Redox measurements and additional chemical treatment phenotypes.

(a and b) GSH (a), GSSG (b) measurements in WT and the *dpe2* mutant. Data are presented as means ± s.e.m. Six biological replicates were performed for each sample. Individual columns were compared using one-way ANOVA with Tukey's post-hoc, different lowercase letters indicate statistical significance at $p < 0.05$. (c) Representative plant phenotypes treated with different chemicals in the absence of SA induction and under different relative humidity (RH) conditions.

bioRxiv preprint doi: https://doi.org/10.1101/2025.10.13.682132; this version posted October 14, 2025. The copyright holder for this preprint (which was not certified by peer review) is the author/funder, who has granted bioRxiv a license to display the preprint in perpetuity. It is made available under aCC-BY 4.0 International license.

The treatment conditions are the same as in Figure 5B. (d) Quantification of the death rate for the plants in (c). (e) Representative plant phenotypes treated with mock or SA plus different chemicals. Plants grown for 14 days under LD cycles were treated with mock or SA plus mock, tunicamycin (TM), 4μ8C, cycloheximide (CHX) or MG115. After all treatments, plants were kept in DD for 72 h before imaging. (f) Quantification of the death rate for the plants in (e). (g) Representative plant phenotypes treated with SA active analogues [2,6-dichloropyridine-4-carboxylic acid (INA) and 2,1,3-benzothiadiazole (BTH)] or inactive analogues [2,4-dihydroxybenzoic acid (2,4-DHBA), benzoic acid (BA), and 3-hydroxybenzoic acid (3-HBA)]. Plants were grown and imaged as in (e). (h) Quantification of the death rate for the plants in (g). Scale bar = 0.5 cm.
